# Angiographic findings in patients with Non-ST-elevation myocardial infarction

**DOI:** 10.12669/pjms.40.10.9499

**Published:** 2024-11

**Authors:** Ikramullah, Farooq Ahmad, Anwarul Haq, Sher Wali Khan

**Affiliations:** 1 Ikramullah, Associate Professor, Cardiology Unit, Lady Reading Hospital, Peshawar, Pakistan; 2 Farooq Ahmad Assistant Professor Cardiology Unit, Khyber Teaching Hospital, Peshawar, Pakistan; 3 Anwarul Haq Residents, Cardiology Unit, Lady Reading Hospital, Peshawar, Pakistan; 4 Sher Wali Khan Residents, Cardiology Unit, Lady Reading Hospital, Peshawar, Pakistan

**Keywords:** Coronary angiography, NSTEMI, OCA, PCI, TVCAD

## Abstract

**Background & Objective:**

Acute coronary syndrome (ACS) can be divided into subgroups of ST-segment elevation myocardial infarction (STEMI), Non-ST-segment elevation myocardial infarction (NSTEMI), and unstable angina (USA). Patients diagnosed with NSTEMI are either treated conservatively or early invasive strategy is adopted. In NSTEMI it is believed that either one or more vessel is transiently occluded or that the blood flow is critically reduced in a patent vessel i.e. subtotal occlusion. This study was conducted to find the angiographic characteristics of NSTEMI patients undergoing coronary angiography.

**Methods:**

This study was conducted in Department of Cardiology, Lady Reading Hospital Peshawar. It was Cross Sectional Study. Data was collected from 8th November 2022 to 8th May 2023. Consecutive non-probability sampling technique was used to collect data from the patients.

**Results:**

Total of 300 patients were studied. The mean age was 56.95 years ±1.176 standard deviation. Male patients were 201 (67%) and remaining were female. It was found that 51(17%) of total NSTEMI patients were having totally obstructed coronary artery (OCA) in one or more coronary arteries. In NSTEMI, non-obstructive CAD (<50%) were found in 81 (27%) of patients whereas 219 (73%) were having significant CAD. It was found that 40% were having single vessel CAD, 32% were having double vessel CAD and 28% were having triple vessel CAD. TVCAD was present significantly higher in advance age, Diabetes mellitus, hyperlipidemia and family history of CAD.

**Conclusion:**

Patients having NSTEMI are usually having multiple risk factors and having usually severe and multivessel CAD.

## INTRODUCTION

Acute coronary syndrome (ACS) is divided into ST-segment elevation myocardial infarction (STEMI), Non-ST-segment elevation myocardial infarction (NSTEM1), and unstable angina.^1^,^2^ NSTEMI is the most common presentation of ACS and the leading cause of hospital admissions.^2^-^4^ Some Studies show that 13.9% of patients diagnosed as acute myocardial infarction are NSTEMI.^5^ In the Middle East including Egypt, 64% of ACS patients present with NSTEMI and 36% present with STEMI according to the results of the ACCESS registry.^6^ Over the last decade, incidence of NSTEMI has markedly increased and associated with lower short-term mortality compared with STEMI patients, while long term mortality is higher than STEMI patients.^7^

Patients diagnosed with NSTEMI are either treated conservatively or early invasive strategy is adopted.^8^ In NSTEMI it is believed that either one or more vessel is transiently occluded or that the blood flow is critically reduced in a patent vessel i.e. subtotal occlusion.^9^ In addition there is lack of evidence in favor of doing emergency angioplasty in patients with NSTEM1.^10^,^11^ For these reasons, according to AHA guidelines doing emergent angiography and angioplasty is not mandatory in all patients with NSTFMI.^12^

Patients who presented with NSTEMI, myocardial function deteriorates and the damage becomes irreversible as the time to angiography and re-vascularization increases, especially in those who have occluded coronaries on angiogram. In clinical practice, electrocardiogram (ECG) criteria are used to differentiate STEMI from NSTEMI, but ECG has a very low sensitivity in detecting acute myocardial infarction and thus occlusion of a coronary vessel.^13^ Both STEMI and NSTEMI can present with severe or complete obstructive coronary artery disease^14^ and various studies show that in NSTEMI, angiography may reveal totally occluded coronary arteries (OCAs), further reinforcing that coronary artery occlusion can occur despite the absence of ST elevation in ECG.^4^ Previous studies have shown that nearly 25% of NSTEMI patients present with a totally occluded coronary artery, and two-thirds of the occlusions are already collateralized at the time of angiographic examination.^15^,^16^

A study done in Karachi Pakistan by Aijaz S et al.^17^ shows that patients who underwent coronary angiography for NSTEMI, 30% had single-vessel CAD and 60% had multi-vessel coronary involvement. Regarding vessel involvement, it was found that, Right Coronary artery (47.6%) was the most frequently involved vessel followed by Left Anterior Descending artery (43.7%) and left circumflex artery (38.3%).^17^

There is no study so far done in KPK on this topic and this study will add valuable data in this part of the world with different ethnic background. This study will also help to find the severity of coronary artery disease in term of its distribution and number of coronaries involved. This will help to identify the severity of diseases in these patients. It will help the physicians to know the grievance of this issue, treat more aggressively these patients and identify high risk patients early so they can be timely managed with early invasive strategy. Our objective was to find the angiographic characteristics of NSTEMI patients undergoing coronary angiography

### Operational definitions:

### NSTEMI

NSTEMI is defined as typical chest pain with ST depressions >l mm or T-wave inversions >l mm and positive Trop-T test.

### Significant coronary artery disease:

Disease was considered significant, if it was more than 50% in any coronary artery (significant obstruction is different from severe stenosis which should be more than 70%). Less than 50% were taken as non-obstructive coronary artery disease.

### Obstructive coronary artery (OCA):

An OCA was defined as presence of a lesion with 100% stenosis in one or more major coronary vessels.

## METHODS

It was cross sectional study conducted at Department of Cardiology, Lady Reading Hospital Peshawar. Study was conducted from 8th November 2022 to 8th May 2023. Sample size was 300. It was calculated using WHO formula for sample size determination in health studies based on, Confidence level of 95%, and margin of error 5%. Sampling technique was Consecutive non-probability sampling technique.

All patients age above 18 years presented with NSTEMI and going for coronary angiography was included in the study. Patients having previous history of Stroke, Myocardial infarction, CABG, renal impairment and previous PCI were excluded from study as these may produce bias in the study results.

### Ethical Approval:

The study was conducted after getting approval from hospital ethical and research committee reference number (302/LRH, dated May 4, 2022). The patients meeting the inclusion criteria were recruited in the study after taking written informed consent. The purpose of the study and the benefits and risks were explained to all the recruited patients.

All patients were managed according to the standard ACS protocol. All patients had undergone a detailed history and clinical examination. Biochemical workup was carried out for each patient including complete blood count, serum electrolytes, RFTs, Lipid profile, RBS, HBA1C, LFTs, ECG and echocardiography for patient management, candidacy for intervention and post intervention care. Angiography was performed by interventional fellow with supervision of consultant interventional cardiologist.

### Data analysis:

Data were analyzed using SPSS Version 23.0. Mean and standard deviation were calculated for quantitative variables like age and BMI. Frequencies and percentages were calculated for categorical variables like gender. Chi square test was used for comparing categorical variables whereas student T test was used for quantitative variables. P-value less than 0.05 was considered significant. All the results were presented in the form of tables.

## RESULTS

Total of 300 patients were studied. The mean age was 56.95 years ±1.176 Standard deviation. Male patients were 201 (67%) and remaining were female. Base line characteristics of study population is shown in Table-I.

**Table-I:** Base line characteristics of study population.

Risk factors	Number	Percentage
Gender (M)	201	67
Diabetes	135	45
Hypertension	186	62
Hyperlipidemia	99	33
Family History of CAD	90	30
Smoking	72	24
Obesity (BMI >25)	195	65
Age above 40 years	250	83%

Regarding coronary artery disease, number and types of coronary arteries were studied. It was found that 51(17%) of total NSTEMI patients were having totally obstructed one or more coronary arteries. In NSTEMI, non-obstructive CAD (<50%) were found in 81 (27%) of patients whereas 219 (73%) were having significant/obstructive CAD. In these 219 patients, 51 patients were having total occlusive coronary artery (OCA) and remaining 168 patients were having significant CAD but non-OCA. Furthermore, it was found that 40% of patients were having single vessel CAD (SVCAD), 32% were having double vessel CAD (DVCAD) and 28% were having triple vessel CAD (TVCAD), (Table-II).

**Table-II:** Coronary artery distribution in NSTEMI patients.

Severity of CAD	Type of coronary artery disease	Number of patients	Percentage
SVCAD		120	40%
	LCX	50	41.66%
	LAD	42	35%
	RCA	28	23.33%
DVCAD		96	32%
	LCX+LAD	40	41.6%
	LCX+RCA	30	31.2%
	LAD+RCA	26	27.08
TVCAD		84	28%
LMS involvement		45	15%

Left main disease with or without involvement of other vessels was found in 15% of patients. Regarding coronary artery distribution in SVCAD, LCX was involved in 42% of cases, LAD was involved in 35% cases and RCA was involved in 23% cases. Furthermore, it was also found that TVCAD is significantly higher in patients having age more than 40 years, Diabetes mellitus, hyperlipidemia and family history of CAD (Table-III).

**Table-III:** Association of TVCAD with risk factors.

Risk factors		TVCAD present N=84	TVCAD absent N=216	P-value
Age	<40y	10	40	P=.04
	>40 y	74	176	
Gender	Male	53	148	P=.7
	Female	31	68	
Hypertension	Yes	52	134	P=.08
	No	32	82	
Smoking	Yes	50	22	P=.03
	No	34	194	
Family history of CAD	+ve	60	30	P=.02
	-ve	24	186	
Hyperlipidemia	yes	51	48	P=.05
	No	33	168	
Diabetes	Yes	135.69	66	P=.001
	No	15	150	
BMI		195.70	125	P=.07
		14	91	

## DISCUSSION

This study is done in tertiary care hospital with well-organized Cath lab facility and 24/7 Primary PCI services. Previously NSTEMI patients were less commonly managed with early invasive strategy. Most of NSTEMI patients in our setup was managed with conservative strategy. With more trained intervention team and Cath facilities more and more of these patients are now going for coronary angiography and more is known now about behavior and nature of NSTEMI patients in our local population.

Our study shows that most of our patients presented for NSTEMI having high risk characteristics. Majority of patients are having multiple major risk factors. Diabetes, hypertension, obesity and hyperlipidemia were very common. Regarding severity of CAD, it was found that 17% of our patients were having OCA. Similar findings of 20% were observed by Ayad SW et al.^18^ Another study conducted by Shin DI et al.^16^ showed that 25% of NSTEMI patients are having OCA. In fact, these findings are contrary to our previous belief that OCA is only seen in STEMI patients. Same interested findings were also observed in our data. A higher incidence of OSA 39% were recorded by Aijaz S et al.^17^ in their study but their study population was very high risk group which may be the reason of this high findings of OCA in their study.

In our study significant CAD (>50%) were found in 73% of patients. These findings are very close to one found by Linde JJ et al.^19^ They found that 68% patients undergoing coronary angiography with NSTEMI is having severe CAD. This shows that most of NSTEMI patients having severe CAD. We found that SVCAD is present in 40% of patients whereas 60% patients are having more than single vessel disease. Similar high findings of severe CAD were noted in other studies in patients who presented with NSTEMI. Aijaz S et at.^17^ found that 60% of NSTEMI patients having multivessel CAD. In another study conducted by Ayad SW^18^, it was found that 71% of patients having more than one vessel CAD who were admitted as NSTEMI and were shifted to cath as early invasive strategy. Linde JJ^19^ et al found in their study that 63% of patients having more than one vessel CAD in patients admitted as NSTEMI.

Moreover, TVCAD was 28% in our study population and similar higher results are recorded by Baligar BD et al who found that 24% of their patients presented as NSTEMI were having TVCAD.^20^ All these findings are very close to our findings and shows how severe and aggressive CAD is noted in patients with NSTEMI. Above findings shows that NSTEMI patients are usually having high risk features and having involvement of multivessel and sever disease. These patients also having large proportion of totally occluded coronary arteries.

### Limitation:

Patients who are usually having low TIMI score and were stable were not proceeded for coronary angiography and that’s the main limitation of our study and the sample may be not truly representative of actual population and the findings can’t be truly generalized on whole NSTEMI population. Also, patients who were having previous CABG, PCI were excluded from the study and it may be effecting on our true study results regarding NSTEMI. Also contrary to STEMI, all NSTEMI patients are not referred to tertiary care hospital and usually managed conservatively which could have effect on our true results and findings.

## CONCLUSION

Patients having NSTEMI are usually having multiple risk factors and having usually severe and multivessel CAD.

### Author`s Contribution:

**I:** Study design, concept, statistics, and final approval.

**FA:** Data analysis and interpretation, final approval and literature search.

**AH:** Data collection, Critical Review and final approval

**SWK:** Data collection, literature search, Review.

All authors have read and approved the final manuscript. They are also responsible and accountable for accuracy and credibility.
